# Improving the Neural Segmentation of Blurry Serial SEM Images by Blind Deblurring

**DOI:** 10.1155/2023/8936903

**Published:** 2023-01-17

**Authors:** Ao Cheng, Kai Kang, Zhanpeng Zhu, Ruobing Zhang, Lirong Wang

**Affiliations:** ^1^School of Electronic and Information Engineering, Soochow University, Suzhou 215009, China; ^2^Jiangsu Key Laboratory of Medical Optics, Suzhou Institute of Biomedical Engineering and Technology, Chinese Academy of Sciences, Suzhou 215163, China; ^3^Etiometry, Inc, 280 Summer St Fl 4, Boston, MA 02210, USA; ^4^Department of Neurosurgery, The First Hospital of Jilin University, Changchun, Jilin 130021, China; ^5^Institute of Artificial Intelligence, Hefei Comprehensive National Science Center, Hefei 230088, China

## Abstract

Serial scanning electron microscopy (sSEM) has recently been developed to reconstruct complex largescale neural connectomes, through learning-based instance segmentation. However, blurry images are inevitable amid prolonged automated data acquisition due to imprecision in autofocusing and autostigmation, which impose a great challenge to accurate segmentation of the massive sSEM image data. Recently, learning-based methods, such as adversarial learning and supervised learning, have been proven to be effective for blind EM image deblurring. However, in practice, these methods suffer from the limited training dataset and the underrepresentation of high-resolution decoded features. Here, we propose a semisupervised learning guided progressive decoding network (SGPN) to exploit unlabeled blurry images for training and progressively enrich high-resolution feature representation. The proposed method outperforms the latest deblurring models on real SEM images with much less ground truth input. The improvement of the PSNR and SSIM is 1.04 dB and 0.086, respectively. We then trained segmentation models with deblurred datasets and demonstrated significant improvement in segmentation accuracy. The A-rand (Bogovic et al. 2013) decreased by 0.119 and 0.026, respectively, for 2D and 3D segmentation.

## 1. Introduction

The SERIAL scanning electron microscope (SEM) is to date the solely available technique to resolve the details of the large-scale neural circuits of animal's nervous system at nanometer resolution, in which the brain's behavioral repertoire and cognitive abilities are embedded[1]. This approach is largely built on high-throughput automatic acquisition and deep learning-based segmentation of unprecedented amounts of serial SEM image data [2]. However, during the often months-long acquisition, image blurring occurs at a significant frequency, mostly due to improper autofocusing and autostigmation and tremendously undermines the subsequent segmentation accuracy. For a routine serial SEM imaging of thousands of sections with 10–1000-fold more tiles, it is simply unrealistic to revisit and reimage every blurry tile. In addition, blurry SEM images also hamper comprehensive preprocessing such as stitching, alignment, and manual annotation. Therefore, a postimaging method to restore the blurry images to high sharpness without disturbing the acquisition is highly desirable.

Conventional methods such as linear [3] or nonlinear filters [4] had been explored to restore the latent high quality images by deconvolutional approaches. However, it cannot be generalized for practical applications, as they require prior knowledge of the blur kernel. Simplified assumptions of the kernel model usually limit their performance on real examples, where real blur models are much more complicated than assumptions.

Recently, deep-learning-based methods have been proven capable of restoring blurry natural images to high quality ones [5–7]. A few algorithms [5, 8] based on the coarse-to-fine strategy were able to reconstruct fine level details against irregular shapes. Adversarial learning was further adopted [9] to learn the pixel spatial distribution for better noise reconstruction. Theoretically, these supervised learning algorithms developed for natural image restoration can be implanted to deblur the SEM image datasets of brain connectome, which own highly irregular morphological features at vastly diverse scales, and complex noise distribution at high frequency. However, in practice, a real SEM dataset usually does not contain paired clear and blurry images of the same field of view, which are required as training datasets. Moreover, SEM images of the brain are highly heterogeneous from one tissue sample to another. Therefore, the shortage of such training dataset greatly limits the performance of supervised learning methods on brain SEM image deblurring and further reduces the accuracy of segmentation of neural structures.

**Figure 1:**
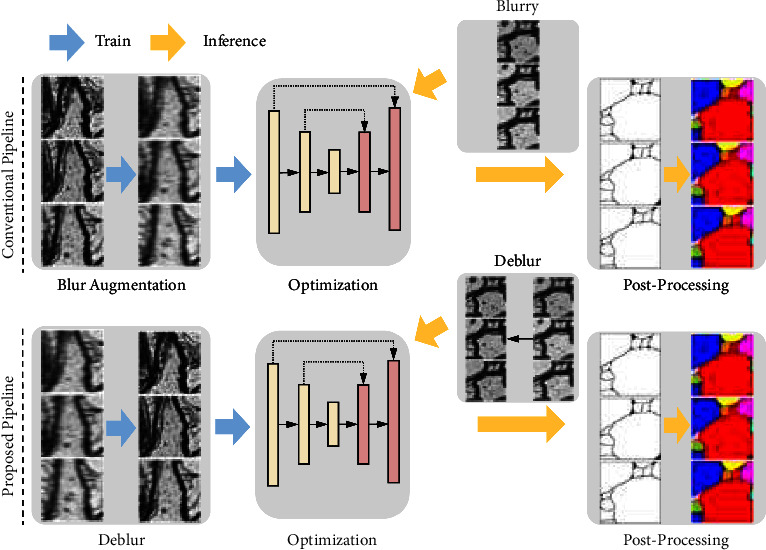
Illustration of the segmentation pipeline. The conventional pipeline uses blur augmentation to improve the model performance against blurry effects. The proposed pipeline feeds deblur images into the segmentation model.

In this work, we propose a semisupervised learning guided progressive decoding network (SGPN) to address this issue. In the network, the generator implements rich, high-resolution features through several hybrid feature extractors (HFEs) in order to progressively discern the features from less informative interval regions such as the cytoplasm or the vascular lumen. Additionally, the proposed semisupervised learning enhances the deblurring performance with limited supervised training data, promoting the generalizability on blurry EM images across different brain samples. We then demonstrate that the accuracy of subsequent instance segmentation is greatly improved with deblurred real SEM image datasets. The proposed method is evaluated on three corpus callosum SEM image datasets, M1, M2, and M3, from different mice and compared with a number of recently reported deblurring methods. As shown in Figure 1, we compare the state-of-the-art deblurring methods on the proposed pipeline to demonstrate the improvement on the segmentation task.

To summarize, this paper makes the following contributions:

1. A novel architecture, semisupervised learning guided progressive decoding network (SGPN), is proposed to restore clear SEM images from blurry ones and therefore to increase subsequent segmentation accuracy.
2. In the SGPN, we designed a hybrid feature extractor (HFE) to optimize the representation of fine features at high resolution and minimize information loss during cross-scale decoding. By doing so, irrelevant features in less informative regions become less significant in learning.
3. The semisupervised learning is joined by adversarial learning with differentiable augmentation to offer the deblurring method higher robustness across image datasets from different brain samples.

The rest of the paper is organized as follows. A few related works are reviewed in Section 2, followed by the details of the proposed method in Section 3, experimental results are presented in Section 4, and eventually, Section 5 concludes the paper with a brief summary.

## 2. Background and Related Work

A blurry EM image *z* can be modeled as the convolution of a clean image *x* with a blur kernel *k*, as

(1) $$\begin{array}{c} z=k*x+\eta \text{,} \end{array}$$

where ^*∗*^ denotes the convolution operation and noise *η* is added. Given *z*, there are several methods that can obtain the underlying clear image *x*. Early work used image priors such as total variation [10, 11] and L0 gradients [12]. These methods, however, cannot be generalized for practical applications as they require prior knowledge of the blur kernel.

Deep-learning-based methods have recently been developed to restore blurry images to clear ones [5, 8, 9, 13–15]. Sun et al. [13] and Chakrabarti [14] reported CNN-based methods to remove motion blur with an unknown kernel. A multiscale CNN with a coarse-to-fine strategy was then developed by Nah et al. [5]. This approach preserves fine-grained detailed information as well as long-range dependency from coarser scales. Tao et al. [8] added encoding, decoding, and ConvLSTM components into a scale-recurrent network to improve computational and statistical efficiency. Furthermore, for blurry images acquired by SEM, a coarse-to-fine strategy was adopted for deblurring [6] and achieved better performance than conventional methods such as the Wiener filter [16] and Richardson–Lucy algorithm [17]. Tsai et al. [18] utilizes nonlocal self-attention design to disentangle blur patterns of different magnitudes and orientations with different receptive fields. However, the limited constraints of CNN-based model will cause the oversmoothing on the restoration results since the output of each pixel is the average value of possible predictions. Kupyn et al. proposed DeblurGAN [15] and DeblurGAN-V2 [9] to obtain clear images through adversarial training for more realistic pixel spatial distribution. Dong et al. [19] utilized a generative adversarial network to deblur microscopic images. These approaches can reconstruct most high-frequency information since generative adversarial network (GAN) architectures focus on the source domain and target domain. However, SEM images of biological specimens, especially the nervous tissues, mostly have highly irregular structural patterns spanning several orders of magnitude in dimension and are extraordinarily heterogeneous across different samples. The aforementioned supervised methods that are highly dependent on the available training dataset would only achieve poor results in deblurring such biological SEM images when the training dataset is insufficient or from dissimilar specimens. Thus, it is essential to resort to semisupervised learning.

Recently, semisupervised learning with GAN has demonstrated outstanding performance on several tasks [20–23]. In practice, these methods resorted to a limited number of image pairs for supervised learning and utilized unlabeled images to provide additional unsupervised constraints. In the segmentation task, Huang et al. [22] used a discriminator to measure the confidence score with unlabeled images and its inferences. In order to provide additional constraints, dual stream semisupervised learning for semantic segmentation model through a GAN branch and a multilabel mean teacher (MLMT) branch, respectively, was proposed by Mittal et al. [23]. Unlike segmentation task, image restoration task optimized the whole image domain mapping instead of the inference domain. Li et al. [24] proposed to exploit the properties of real-world clean images via pixel spatial distribution, sparsity of dark channel, and gradient priors. However, this approach of unsupervised branches only used global prior, which neglect the domain-specific knowledge. In [20], You et al. proposed a semisupervised GAN-Cycle method to obtain higher-resolution images. Moreover, based on GAN architecture, they developed a nonlinear end-to-end mapping from noisy input images to denoised and deblurred outputs. Yang [25] introduced a semisupervised method to learn the marginal distribution of each modality based on unpaired images by minimizing the Wasserstein distance between the distributions of real and fake images.

These methods present a GAN-based architecture and have better performance when implementing semisupervised learning. In practice, we found that constraining domain mapping of unsupervised images to real data distribution [20, 25] may yield features to false positive distribution. More specifically, the generator output discriminative spatial distributions between supervised datasets and unsupervised datasets because it utilizes the discriminator to identify the dataset instead of data distribution. This degeneracy of mapping exhibited on the rapid convergence on unsupervised data distribution, while supervised data are adversarial converging. In contrast to these methods, we make a GAN architecture with semisupervised learning that the unsupervised branch is constraining the domain map into deblurred results from supervised images instead of clear images.

## 3. Proposed Method

Our model is a GAN-based, semisupervised learning architecture. We implement multiscale CNN with a progressive decoding strategy as the generative network (G-Net) and use semisupervised learning through the discriminator network (D-Net) to increase the generalizability of the deblur method. Furthermore, for smoother convergence of pixel spatial distribution, differentiable augmentation [26] is added for the images that are fed into the D-Net.

### 3.1. Generative Network (G-Net)

**Figure 2:**
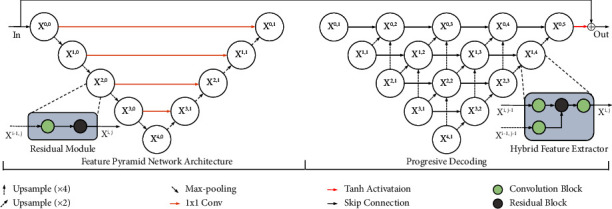
Illustration of our G-Net. *i* and *j* on *X*^*i,j*^ represents the scale index and processing index. On the left-hand side is the feature pyramid structure with residual blocks *X*^*i,j*^ on each scale. For the skip connection, we propose 1 × 1 convolution layers to reduce the feature channels and sum with decoded features for coarse decoding. On the right-hand side is our proposed progressive fine decoding structure through HFE. Note that *X*^*i,*1^ do not implement any processing, they are coarse decoded features from left. Moreover, we apply the Tanh activation function to the output from *X*^0,5^ and the element-wise sum between output and input as global residual connection to obtain the final result.

As shown in Figure 2, we build our backbone network using feature pyramid architecture [27] with convolution block and light-weight residual block [28] on each scale of the encoder and decoder. Additionally, on the right part of Figure 2, the progressive decoding strategy is elucidated, which contains repeated hybrid feature extractors (HFEs) to exploit coarse and medium resolution information while preserving high-resolution information.

#### 3.1.1. Hybrid Feature Extractors

**Figure 3:**
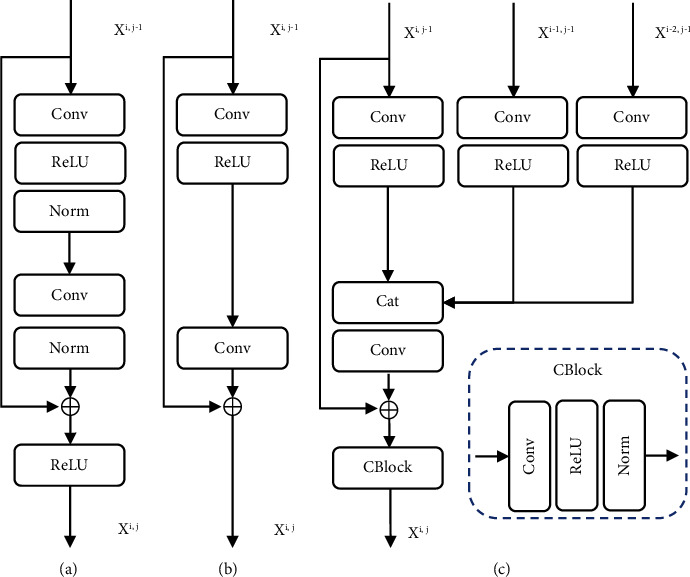
Illustration of our hybrid feature extractor. (a) Common residual block; (b) light-weight residual block corresponding to the study by Lim et al. [28] and Nah et al. [5]; (c) the proposed extractor with external features from other scales *X*^*i*−1, *j*^, *X*^*i*−1,*j*^. They are concatenated (cat) inside the light-weight residual block.

As shown in Figure 3, comparing with the residual block method (Figure 3(a)) [29], Lim et al. [28] removed two normalization layers and one activation function (Figure 3(b)). For the image reconstruction tasks, the light-weight residual block has similar performance but a lower computation cost. We found multiscale CNN-based information loss amid decoding. Therefore, we keep the light design of residual block but add extra lower-scale features. For coarse-to-fine resolution feature reconstruction, we assemble residual blocks at *X*^*i*,*j*^ scale with features from upsampled *X*^*i*−1,*j*^ and *X*^*i*−2,*j*^, *i*∈ (0, 1, 2, 3, 4), *j* ≤ (5−*i*) to enrich high-resolution representation. We add skip connections between *X*^*i*,*j*−1^ and convolution results after the concatenation step. Furthermore, a convolution block is inserted after the element-wise sum step. The goal is to progressively decode the features from low to high resolution, maximizing the efficiency and representation of the lower-scale features in the multiscale CNN architecture.

### 3.2. Discriminative Network (D-Net)

Conventional deep learning-based image enhancement methods may cause oversmoothing and extra blurring artifacts because they output the average prediction for each pixel. Here, we introduce a discriminator *D* and a generator *G* in our deblur framework for better spatial pixel distribution. We aim to solve an adversarial min-max problem *V* (*D*, *G*), which can be described mathematically as follows:

(2) $$\begin{array}{c} \min_{G}\max_{D}V\left(D,G\right)=E_{x∼p_{\text{data}}\left(x\right)}\left[\log\;\left(D\left(x\right)\right)\right]+E_{z∼p_{z}\left(z\right)}\left[\log\;\left(1-D\left(G\left(z\right)\right)\right)\right]\text{.} \end{array}$$

The generative network *G* aims to fool the discriminator *D* by misleading *D* to output higher score for the fake inputs, while the discriminator *D* tries to give a higher score for clear images and a lower score for denoised images. With this alternative approach, our generator *G* learns to restore images similar result to the clear ones so that it becomes difficult for the discriminator *D* to distinguish.

We adopt the idea of least squares GAN's [30] discriminator, which provides a smoother and nonsaturating gradient to fix the vanishing gradients and stabilize the training. We suppose LSGAN uses the *a* − *b* coding scheme for the discriminator, where a and *b* are the labels for fake data and real data, respectively. In addition, *c* denotes the value that *G* wants *D* to believe for fake data. Then, we obtain the following objective functions:

(3) $$\begin{array}{c} \min_{D}V\left(D\right)=\frac{1}{2}E_{x∼p_{\text{data}}\left(x\right)}\left[{\left(D\left(x\right)-b\right)}^{2}\right] \end{array}$$

(4) $$\begin{array}{c} \min_{G}V\left(G\right)=\frac{1}{2}E_{z∼p_{z}\left(z\right)}\left[{\left(D\left(G\left(z\right)\right)-c\right)}^{2}\right]\text{.} \end{array}$$

In order to make *G* generate similar spatial distribution with *D*, we set *c* = *b* = 1 and *a* = 0 by using the 0 − 1 binary coding scheme in equations (3) and (4).

### 3.3. SGPN

**Figure 4:**
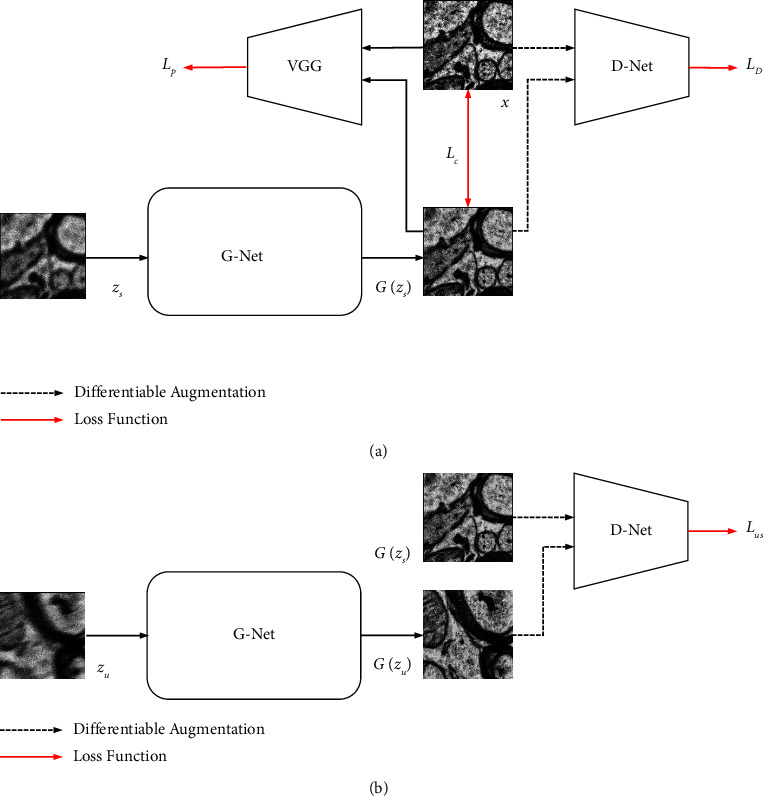
Illustration of model training structure. Firstly, the supervised image *z*_*s*_ and unsupervised image *z*_*u*_ are fed into the generator. For the supervised learning, perceptual loss, adversarial loss, and content loss are calculated through VGG19, discriminator, and the supervised image pair, respectively. For the unsupervised learning, we use the measurement score from the discriminator to minimize the differences between *G*(*z*_*s*_) and *G*(*z*_*u*_). Note that all the images that are fed into D-Net are processed with differentiable augmentation.

During the training of SGPN, we input paired supervised images [*z*_*s*_, *x*] and unsupervised images *z*_*u*_, respectively. As shown in Figure 4, both blurry images are fed into G-Net to obtain the deblurred results ${\hat{z}}_{s}$ and ${\hat{z}}_{u}$. Then, the discriminator measures the distribution score for *T*(${\hat{z}}_{s}$) and *T*(${\hat{z}}_{s}$) where *T*(·) represents differentiable augmentation [26]. Moreover, we calculate the textual and perceptual difference between paired images ${\hat{z}}_{s}$ and *x* from VGG-Net [31]. The deblur network G-Net is trained with four losses in total: content loss, perceptual loss, adversarial loss, and unsupervised loss.

#### 3.3.1. Content Loss

We use a robust pixel-wise L1 function as the content loss *ℒ*_*c*_ for our deblurring network.

(5) $$\begin{array}{c} L_{c}={\left\|x-{\hat{z}}_{s}\right\|}_{1}\text{.} \end{array}$$

#### 3.3.2. Perceptual Loss

Inspired by the benefits of the perceptual loss in style transfer [32] and image super-resolution [33] tasks, we use it to provide additional heterogeneous structural features from the pretrained VGG-19 network [31]. We compute the Euclidean loss between model output *G*(*z*_*s*_) and the clear image *x* on the feature maps from conv3 3, where *C*, *H*, and *W* represent channel, height, and width in the following equation:

(6) $$\begin{array}{c} L_{p}=\frac{1}{C*H*W}{\left\|VGG_{19}\left(x\right)-VGG_{19}\left({\hat{z}}_{s}\right)\right\|}_{2}^{2}\text{.} \end{array}$$

#### 3.3.3. Adversarial Loss and Unsupervised Loss

The adversarial loss and unsupervised loss aim to minimize the difference of spatial pixel distribution between the generator's output and the real clear images. The loss terms Ladv and Lus are defined as

(7) $$\begin{array}{c} L_{\text{adv}}=E_{z_{s}∼p_{z}\left(z\right)}\left[{\left(D\left(T\left({\hat{z}}_{s}\right)\right)-1\right)}^{2}\right]\text{,}\\ L_{us}=E_{z_{s},z_{u}∼p_{z}\left(z\right)}\left[{\left(D\left(T\left({\hat{z}}_{s}\right)\right)-D\left(T\left({\hat{z}}_{u}\right)\right)\right)}^{2}\right]\text{.} \end{array}$$

We use the discriminator *D* as a measurement of the reconstruction score. *T*(·) represents differentiable augmentation [26] step of all the images before feeding to D-Net. We conducted color, translation, and cutout augmentation on each image. We also empirically conclude that semisupervised learning through LSGAN can generate higher perceptual quality and overall sharper outputs on human visual perception. Furthermore, we aim to minimize the spatial difference between ${\hat{z}}_{s}$ and ${\hat{z}}_{u}$ instead of real data and ${\hat{z}}_{u}$. This step provides the constraint of learning invalid distributions and features. Because we found that design to converge ${\hat{z}}_{u}$ into real data distribution leads to fast Lus loss convergence and generate invalid distribution since the real and fake data are unpaired.

#### 3.3.4. Overall G-Net Loss Function

The final loss function is composed of three items with different weight values:

(8) $$\begin{array}{c} L_{G}\left(G,D,VGG_{19}\right)=L_{c}\left(G\right)+\lambda_{\text{adv}}L_{\text{adv}}\left(G,D\right)+\lambda_{us}L_{us}\left(G,D\right)\\ +\lambda_{p}L_{p}\left(G,{VGG}_{19}\right)\text{,} \end{array}$$

where *λ*_adv_, *λ*_us_, and *λ*_*p*_ are constant.

#### 3.3.5. D-Net Loss Function

Instead of putting in unpaired images as real and fake images into the discriminator [21, 23], we prefer to use supervised images to maximize the ability to distinguish between the spatial pixel distribution of blurry and clear images. The convolution layer in *D* is set up with 4 × 4 convolution kernel size layer with stride 2. An instance normalization layer and a LeakyReLu activation function are also implemented at each scale. The loss term is defined as

(9) $$\begin{array}{c} L_{D}\left(G,D\right)=\frac{1}{2}E_{x∼p_{\text{data}}\left(x\right)}\left[{\left(D\left(T\left(x\right)\right)-1\right)}^{2}\right]\\ +\frac{1}{2}E_{z_{s}∼p_{z}\left(z\right)}\left[{\left(D\left(T\left({\hat{z}}_{s}\right)\right)\right)}^{2}\right]\text{,} \end{array}$$

where *L_D_* updated with supervised and unsupervised dataset.

## 4. Experiment Results

### 4.1. Experiment Settings

**Figure 5:**
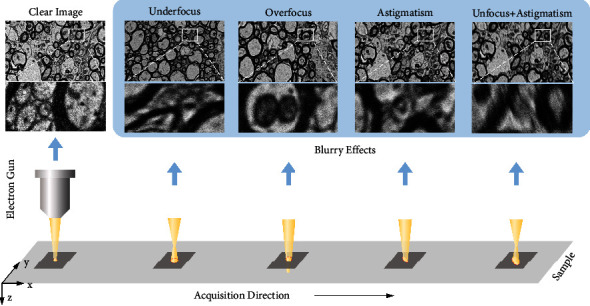
Illustration of our data acquisition. It includes the blurry effects such as underfocus, overfocus, astigmatism, and underfocus + astigmatism.

Sample preparation and EM imaging parameters can be found in Table 1, “Sec” and “Img” means section and image, respectively. M1, M2, and M3 represent different mice. The blurry effects are shown in Figure 5. When acquiring clear images of each section in M1, we purposely generated three types of blurry images of the same area. With 9 sections in M1, we can acquire 9 defocusing images, 9 astigmatic images, and 9 images of both effects. In order to justify the effects of section thickness and sample difference across mice, in M2, we collected 15 sections with 40 nm and 60 nm thickness, respectively. On each section in M2, we only generate one blurry effect while acquiring a clear image. Eventually, we obtained 5 defocused images, 5 astigmatic images, and 5 images of both effects with 40 nm and 60 nm thickness, respectively. Both blurry images in M1 and M2 are acquired by purposely adjusting the objective lens, the stigmators. M3 contains clear and blurry images. However, blurry images in M3 are acquired unintentionally, which means the blurry effects are unknown.

#### 4.1.1. Implementation Details

We split 27 high-resolution EM images from dataset M1 into 1,728 subimages (1 K resolution) and attribute them to training and testing sets at different ratios. Similarly, we prepared 1920 subimages (1 K resolution) from the dataset M2 for quantitative evaluation. Ultimately, we manually annotated three different volume datasets from M3 as M3-1, M3-2, and M3-3 for further segmentation experiments.

We set 3 × 3 as the kernel size of Conv layers with a zero-padding strategy in residual blocks. For training, we set *λ*_adv_, *λ*_us_, and *λ*_*p*_ equals to 0.1, 0.01, and 0.02, respectively. Our model was trained by an SGD optimizer with a warm-up learning rate strategy until it reaches 0.0001. We used PyTorch [34] to implement models on 6 NVIDIA RTX3090 GPUs for training with mini-batch size 2 and 256 × 256 resolution of image as inputs. During the training, we found that implementing an instance normalization layer on each scale of the pyramid structure (Figure 2) led to stable training convergence.

### 4.2. Evaluation

We compare the performance of deblurring methods of the following algorithms: DeepDeblur [5] and SRN [8] with coarse-to-fine optimization, BANet [18] with self-attention design, and the GAN-based architecture DeblurGANV2 [9]. We selected peak signal-to-noise ratio (PSNR) and structural similarity index metric (SSIM) [35] as the criteria for quantitative evaluation.

#### 4.2.1. Deblurring Comparison

**Figure 6:**
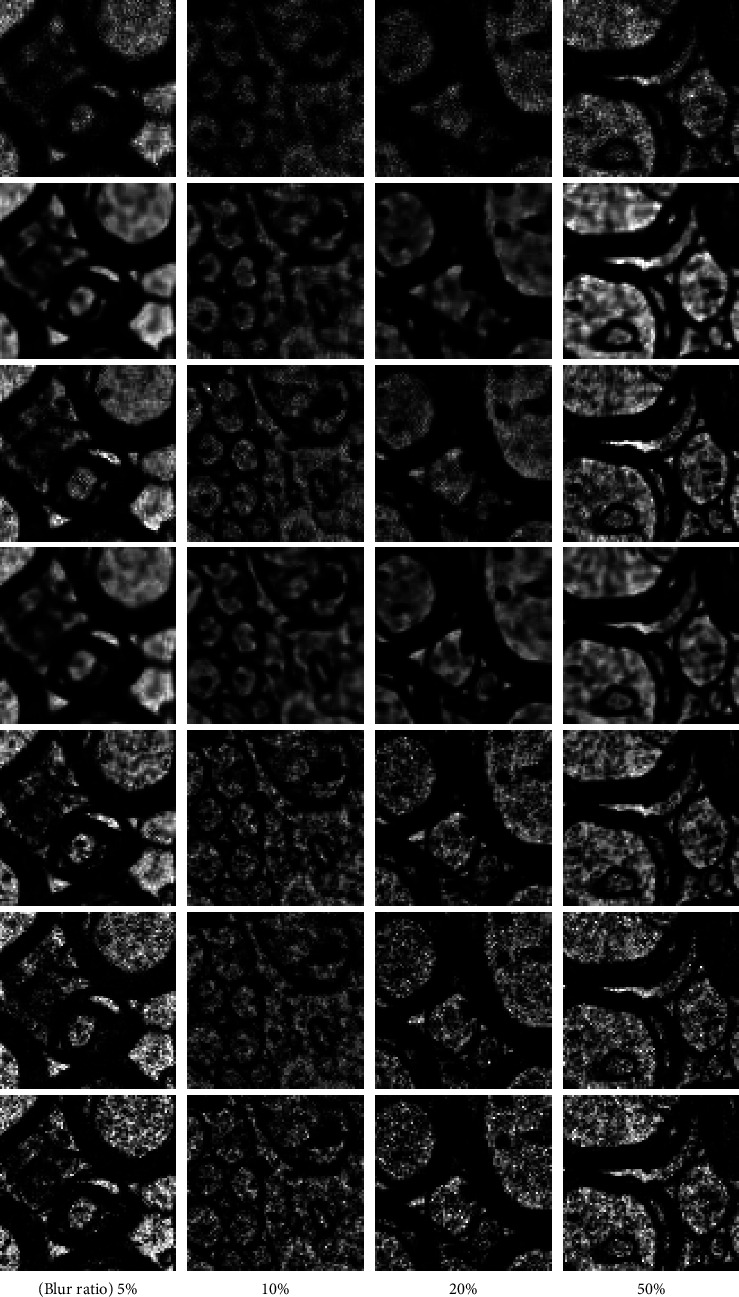
Evaluation results from the M1 dataset. The first to the fourth column represents the 5% training dataset, 10% training dataset, 20% training dataset, and 50% training dataset, respectively. The first and last rows are blurry images and clear images. From the second row to the fifth row represents the deblurring method corresponding to the study by Nah et al. [5], Kupyn et al. [9], Tao et al. [8], Tsai et al. [18], and our proposed method. As shown in this figure, our proposed method can reconstruct myelin sheaths and intracellular features with accuracy.

The evaluation of different deblurring methods with a different rate between training and testing is shown in Figure 6 and Table 2. For this experiment, our semisupervised learning is implemented with the rest of the images from M1. We can clearly observe that the proposed method produces sharper and more accurate reconstructions compared with other methods. When the training dataset is limited, BANet [18] has the best PSNR compared with other methods. Semisupervised learning with a 50% training dataset works well, and our method improves the performance by 2.49 dB and 0.186 on PSNR and SSIM, respectively, while the other best-performing method [18] achieves 2.28 dB and 0.179 on PSNR and SSIM.

**Figure 7:**
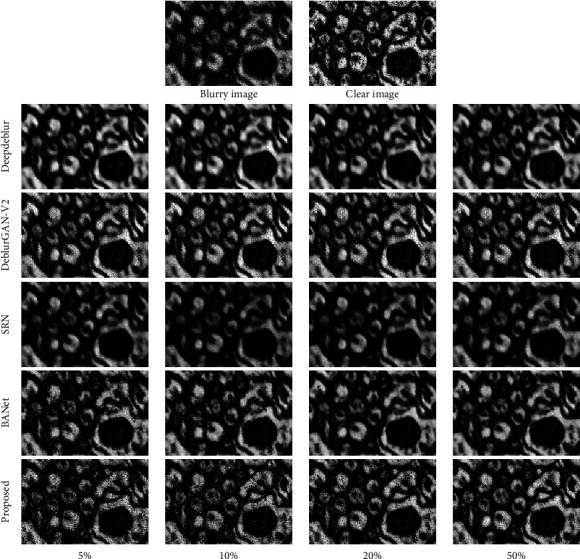
Deblurring results from the M2 dataset. The top row is the blurry EM image and its clear image. The rest are deblurring methods from the study by Nah et al. [5], Kupyn et al. [9], Tao et al. [8], and Tsai et al. [18] and our proposed method. Besides, each column represents the number of training samples from M1. As can be seen from this figure, on the fourth column, deblurring model without adversarial learning causes spatial blur on the whole image.

In addition, we tested the images from dataset M2 to see the generalization of deep-learning-based methods. Noted that M2 was collected on different mice, and it contains 40 nm and 60 nm thickness of sections. In this experiment, the results of the proposed method were acquired by adding the rest of the M2 dataset to the semisupervised learning bank. The quantitative and visual results are shown in Table 3 and Figure 7. As it can be seen from Table 3, although BANet [18] had a similar performance to our method in the previous experiment, our proposed method outperformed other methods with all the different ratios of the training dataset. Because of the proposed semisupervised learning, we can increase the capability of the model's generalization to overcome the challenge of feature gaps between datasets. As shown in Figure 7, our proposed semisupervised learning presents a real-world solution for deblurring cross samples and section thickness.

#### 4.2.2. Deblur Quality

To show the significance of different deblurring methods, we demonstrated 2D segmentation accuracy enhancement through A-rand [36, 37]. We trained a 2D segmentation model to generate binary foreground probability maps and instance contours [38]. For this comparison, we collected 160 clear images from M3 with the resolution 1.5 K × 1.5 K as the training dataset. Since the segmentation model is only trained on clear images, the better the final segmentation, the better the deblurring images are close to the training images. Moreover, for a fair comparison, we implemented the same training augmentation and test-time augmentation for training and inferencing for all the models. We note that the segmentation model is only trained with clear images. Furthermore, we removed blur augmentation, and then we input the deblurred images for testing. After acquiring the inference results from the segmentation model, eventual segmentation results are obtained from the watershed algorithm. Noted that in this experiment, all the adjustable parameters for the postprocessing algorithm are fixed.  Table 4 clearly shows that the deblurred images produced by our method have better cell feature recognition accuracy than the other state-of-the-art methods on the 2D SEM image segmentation task.

Moreover, it demonstrates that a deep-learning-based method can reconstruct most cell structures and have a positive impact on the segmentation model as the model is only trained by clear images. This experiment clearly shows that our method can retain the important features of cells while performing deblurring. This in turn helps in achieving better segmentation results compared to the other methods.

#### 4.2.3. Deblurring-to-Segmentation Pipeline

**Figure 8:**
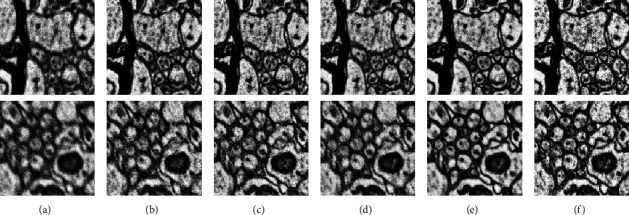
Ablation study with the M2 dataset. (a) Blurry image; (b) adversarial learning (baseline); (c) adding perceptual loss; (d) adding differentiable augmentation; (e) adding semisupervised learning; (f) clear image. First and second rows have section thickness of 40 nm and 60 nm, respectively.

**Figure 9:**
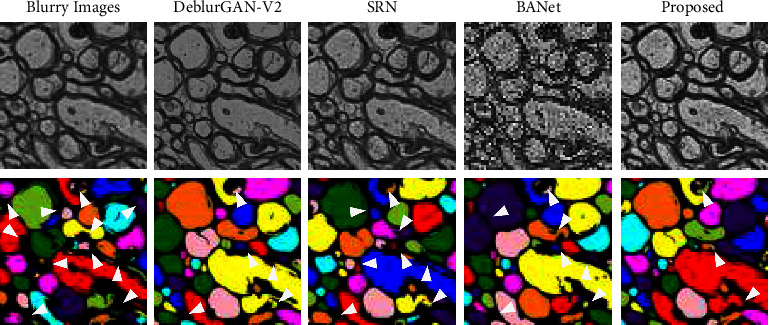
Evaluation results for 2D deblur-to-segmentation approach design. Left column is the blurry image and its segmentation result; SRN and DeblurGAN-V2 is the deblurring method corresponding to the study by Kupyn et al. [9], Tao et al. [8], and Tsai et al. [18], respectively; right column is our proposed deblurring methods and segmentation results. Note that each column represents a different 2D segmentation model. SRN, DeblurGAN-V2, and the proposed method are implemented through deblur-to-segmentation approaches. As shown in this figure, the deblur-to-segmentation approach performs better than a single 2D segmentation model with blur augmentation.

We test the segmentation model's performance when feeding deblurred images into their training, which becomes a deblurring-to-segmentation learning-based method. In this experiment, we only optimize the segmentation model, while the deblurring model remains unchanged. We choose M3-1 as the training dataset, and M3-2 as the testing dataset.  Table 5 clearly shows our method has the highest segmentation accuracy than other deblurring methods, which means our method has richer and more accurate feature information feeding to segmentation training. Moreover, as shown in Figure 8, deblurring-to-segmentation pipeline performs better than the single segmentation model on the 2D segmentation task. Furthermore, the performance on 3D segmentation results is shown in Figure 9. The consecutive images demonstrate that the proposed deblurring-to-segmentation approach can increase segmentation accuracy. Moreover, this experiment provides the solution for real-world defocused EM image segmentation enhancement because this deblurring-to-segmentation method outperforms the single 2D or 3D segmentation model with the blur augmentation method.

#### 4.2.4. Ablation Study

We conduct experiments on two datasets with a 50% training dataset each from M1 and M2 to study the contribution of each component in SGPN. We started with an adversarial learning-based network with the least square adversarial loss. Next, we progressively add components to demonstrate their improvement and estimate the final deblurred image. The results are shown in Table 6. As shown in this table, with the M1 dataset, the introduction of adding the perceptual loss and differentiable augmentation improved the performance by 0.17 dB and 0.033 on PSNR and SSIM, respectively. When evaluating the M2 dataset, the performance improved by 0.15 dB and 0.013 on PSNR and SSIM. When SGPN is trained using LG (*G*, *D*, and VGG19) with the M1 dataset, the ultimate model gains 0.6 dB and 0.055 on PSNR and SSIM compared with adversarial learning. With the M2 dataset, we obtain an enhancement of 0.34 dB and 0.027 on PSNR and SSIM, respectively, compared with the baseline.

**Figure 10:**
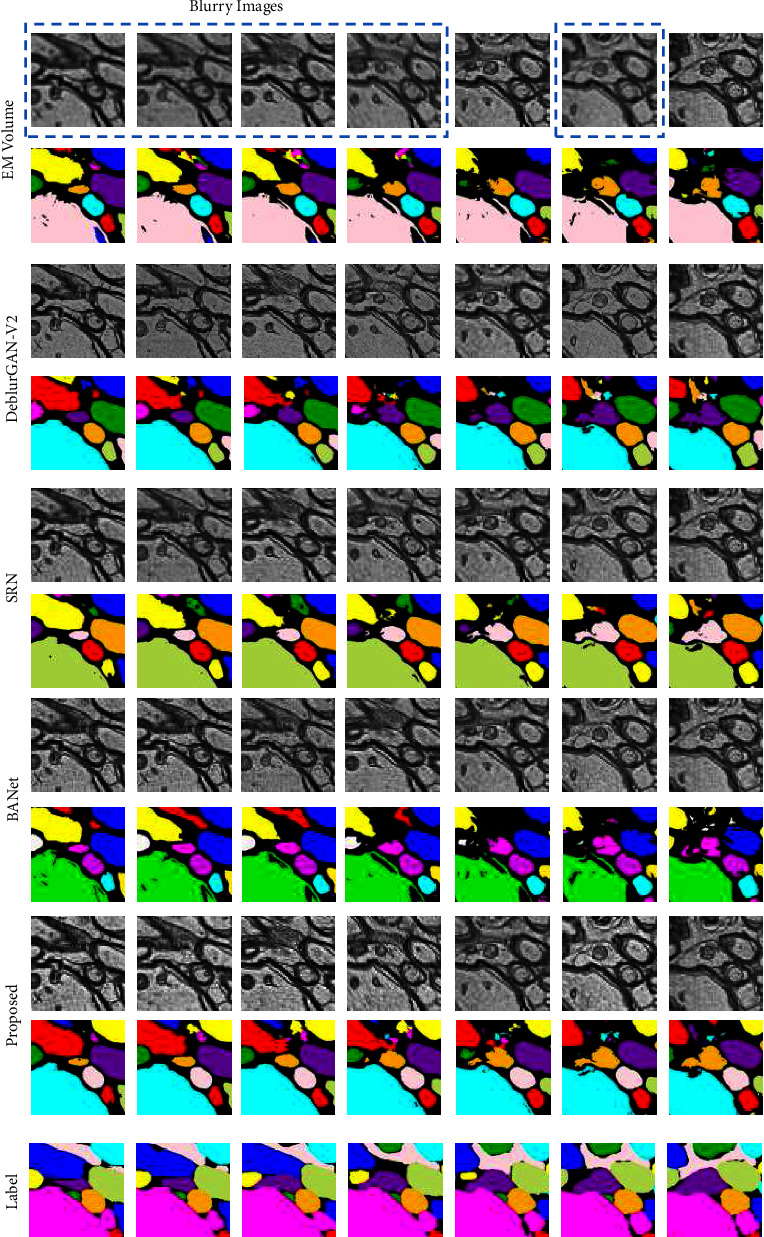
Illustration of consecutive EM images for the training of the 3D segmentation model. EM volume: original EM volume with focused and blur images and its 3D segmentation results; DeblurGAN-V2, SRN, and BANet: deblurring EM images according to the method from Kupyn et al. [9], Tao et al. [8], and Tsai et al. [18], respectively, and its 3D segmentation results; proposed: deblurring EM images according to the proposed deblurring method and our 3D segmentation results; label: annotated ground truth. Focused EM images are not replaced, such as the fifth and seventh columns. The rest of the deblurring images are implemented with different well-performed deblurring methods. Note that deblur-to-segmentation learning-based approaches, GAN-based or not, are better than the single 3D segmentation model with blur augmentation. Our proposed method has better performance on recovery of cell features and spatial noise distribution, leading to higher segmentation accuracy.

Figure 10 demonstrates the reconstructions of axons using the M2 dataset with a sample thickness of 40 nm and 60 nm, respectively. As shown in this figure, our baseline model cannot recover most cell boundaries and fails to reconstruct heterogeneous structures such as mitochondria and vesicles until adding perceptual loss. On the other hand, adding differentiable augmentation for stable adversarial training improves the spatial pixel distribution of cytoplasm and the background region, but it does not provide a stronger capability of distinguishing cell boundaries. Adding semisupervised learning helps SGPN reconstruct qualitatively sharper SEM images with a more accurate cell boundary. Furthermore, it has a positive impact on subsequent segmentation tasks.

### 4.3. Inference Time and Parameters

We profile the parameters of the models and inferencing times in Table 7. In this section, we measure the inference time of a batch with batch size 60. The input size is 256 × 256 for all the compared methods. We observe that the self-recurrent models, DeepDeblur [5], and SRN [8] consume longer runtime than the nonrecurrent methods such as DeblurGAN-V2 [9], BANet [18], and ours. As shown in Table 7, our method runs faster than the DeepDeblur and has fewer parameters than DeblurGAN-V2.

## 5. Conclusion

In this article, we implement augmented semisupervised learning in a GAN where we introduce a network called SGPN, containing hybrid feature extractors (HFE), for EM image deblurring. Compared with existing models, our proposed model can better represent the distinctive spatial distribution of pixels in heterogeneous structural features from microscopic images and is more generalizable across different datasets. It demonstrates superior performance to deblur EM images with different blur effects using limited training samples. We gain 1.04 dB and 0.086 improvements on PSNR and SSIM, respectively, with 50% training dataset of the different samples. Segmentation experiments prove that the proposed model with semisupervised learning, when used as a preprocessing step or deblur-to-segmentation approach, achieves 0.119 and 0.026 decreases in the A-rand score [36, 37] of 2D and 3D neural segmentation tasks. Our work significantly improved the approach of promoting segmentation accuracy by restoring the inevitable blurry images in large-scale serial EM datasets, greatly facilitating the reconstruction of neural connectome maps, an emerging field of tremendous importance to the understanding of brain fundamental principles.

## Figures and Tables

**Table 1:** Mouse brain sample preparation and the parameters for EM image acquisition.

Dataset	M1	M2	M3
Sec thickness	40 nm	40 nm and 60 nm	60 nm
Resolution	4 nm	4 nm	4 nm
EM (zeiss)	GeminiSEM 300	GeminiSEM 300	Supra55
Cropped size	1 K ∗ 1 K	1 K ∗ 1 K	3 K ∗ 3 K
Num of secs	9	15 and 15	59
Total imgs	1728	960 and 960	59 ∗ 3

**Table 2:** PSNR (DB) and SSIM comparison between different deblur methods from the M1 dataset.

Method	5% training		10% training		20% training		50% training	
	PSNR	SSIM	PSNR	SSIM	PSNR	SSIM	PSNR	SSIM
Blurry	14.85	0.159	14.85	0.159	14.86	0.160	14.86	0.160
Nah et al. [5]	16.21	0.258	16.35	0.269	16.51	0.272	16.92	0.286
Kupyn et al. [9]	16.23	0.229	16.30	0.232	16.73	0.268	17.08	0.312
Tao et al. [8]	16.37	0.276	16.49	0.281	16.58	0.288	17.01	0.297
Tsai et al. [18]	**16.53**	0.287	**16.62**	0.298	**16.67**	0.317	17.14	0.339
Proposed	16.48	**0.291**	16.59	**0.304**	16.62	**0.335**	**17.35**	**0.346**

**Table 3:** PSNR (DB) and SSIM comparison between different deblur methods from the M2 dataset.

Method	5% training		10% training		20% training		50% training	
	PSNR	SSIM	PSNR	SSIM	PSNR	SSIM	PSNR	SSIM
Blurry	13.97	0.228	—	—	—	—	—	—
Nah et al. [5]	14.31	0.246	14.42	0.254	14.55	0.261	14.65	0.276
Kupyn et al. [9]	14.38	0.263	14.46	0.269	14.58	0.278	14.72	0.285
Tao et al. [8]	14.45	0.257	14.57	0.263	14.65	0.269	14.74	0.294
Tsai et al. [18]	14.51	0.261	14.63	0.271	14.77	0.282	14.88	0.291
Proposed	**14.77**	**0.275**	**14.86**	**0.289**	**14.92**	**0.290**	**15.01**	**0.314**

**Table 4:** Segmentation accuracy on deblurred images.

Description	2D seg-model
	Arand↓
w/o deblurring	0.459
Kupyn et al. [9]	0.360
Tao et al. [8]	0.373
Tsai et al. [18]	0.357
Propose	**0.335**

**Table 5:** Segmentation improvement based on deblur-to-segmentation learning-based method approach.

Description	2D seg-model	3D seg-model
	Arand↓	
w/o deblurring	0.406	0.252
Kupyn et al. [9]	0.302	0.233
Tao et al. [8]	0.316	0.231
Tsai et al. [18]	0.304	0.232
Proposed	**0.287**	**0.226**

**Table 6:** PSNR (DB) and SSIM comparison of ABLATION study.

Method	M1 dataset		M2 dataset	
	PSNR	SSIM	PSNR	SSIM
Blurry images	14.86	0.160	13.97	0.228
Adversarial learning (baseline)	16.93	0.291	14.67	0.277
+ Perceptual loss	16.98	0.301	14.74	0.283
+ DiffAug	17.10	0.324	14.82	0.290
+ Semisupervised learning	17.53	0.346	15.01	0.304

**Table 7:** Time cost and parameters. Time and params are measured in millisecond (MS) and million (*M*).

	Nah et al. [5]	Kupyn et al. [9]	Tao et al. [8]	Tsai et al. [18]	Proposed
Params	11.69	60.93	7.36	17.98	19.34
Time	20.3	13.6	15.6	17.1	17.4

## Data Availability

The data used to support the findings of this study are available from the corresponding author upon request.
